# Genetically Different Highly Pathogenic Avian Influenza A(H5N1) Viruses in West Africa, 2015

**DOI:** 10.3201/eid2212.160578

**Published:** 2016-12

**Authors:** Luca Tassoni, Alice Fusaro, Adelaide Milani, Philippe Lemey, Joseph Adongo Awuni, Victoria Bernice Sedor, Otilia Dogbey, Abraham Nii Okai Commey, Clement Meseko, Tony Joannis, Germaine L. Minoungou, Lassina Ouattara, Abdoul Malick Haido, Diarra Cisse-Aman, Emmanuel Couacy-Hymann, Gwenaelle Dauphin, Giovanni Cattoli, Isabella Monne

**Affiliations:** Istituto Zooprofilattico Sperimentale delle Venezie, Legnaro, Italy (L. Tassoni, A. Fusaro, A. Milani, I. Monne);; University of Leuven, Leuven, Belgium (P. Lemey);; Veterinary Services Directorate of Ministry of Food and Agriculture, Accra, Ghana (J. Adongo Awuni, V.B. Sedor, O. Dogbey, A.N.O. Commey);; National Veterinary Research Institute, Vom, Nigeria (C. Meseko, T. Joannis);; Laboratoire National d’Elevage, Ouagadougou, Burkina Faso (G. Minoungou);; Ministère des Ressources Animales, Ouagadougou (L. Ouattara);; Direction de la Santé Animale, Niamey, Niger (A.M. Haido);; Ministère des Ressources Animales et Halieutiques, Abidjan, Côte d'Ivoire (D. Cisse-Aman);; Laboratoire Central de Pathologie Animale, Bingerville, Côte d'Ivoire (E. Couacy-Hymann);; Food and Agriculture Organization of the United Nations, Rome, Italy (G. Dauphin);; International Atomic Energy Agency, Seibersdorf, Austria (G. Cattoli)

**Keywords:** Influenza A virus, H5N1 subtype, highly pathogenic avian influenza, HPAI, phylogeny, Africa, viruses, genetic groups, West Africa, respiratory infections

## Abstract

To trace the evolution of highly pathogenic influenza A(H5N1) virus in West Africa, we sequenced genomes of 43 viruses collected during 2015 from poultry and wild birds in 5 countries. We found 2 co-circulating genetic groups within clade 2.3.2.1c. Mutations that may increase adaptation to mammals raise concern over possible risk for humans.

In December 2014, a strain of highly pathogenic avian influenza (HPAI) A(H5N1) virus responsible for deaths among poultry was detected in southwestern Nigeria, specifically in a live bird market in Lagos State (*1*). Since then, other outbreaks have occurred in Nigeria, and the HPAI A(H5N1) virus has also been officially reported in Burkina Faso (February 2015) and Niger, Ghana, and Côte d’Ivoire (April 2015), to date causing the death of ≈1.6 million birds (*2*).

Previous HPAI A(H5N1) epidemics in West Africa occurred in 2006–2008 and involved exclusively viruses of clade 2.2 (*3*). So far, a full-genome characterization is publicly available for only 1 HPAI A(H5N1) virus, collected in Nigeria in early 2015 (*4*) and classified as clade 2.3.2.1c. To our knowledge, this clade has not been previously detected in Africa. Since 2009, this clade has been widely circulating in domestic and wild birds in several countries in Asia (*5*); in 2010, it was reported in Europe (*6*) and in 2014, in the Middle East (*7*). In 2015, clade 2.3.2.1c was detected in rooks, chickens, and dalmatian pelicans in Russia, Bulgaria, and Romania, respectively (*8*). To trace the evolution of HPAI A(H5N1) virus in West Africa, we examined the genetic characteristics of 43 such viruses collected during January–August 2015 in all affected countries in West Africa.

## The Study

From January through October 2015, a total of 248 samples (organ tissue and swab samples) from poultry and wild birds suspected of being infected with HPAI A(H5N1) virus in 6 countries in West Africa were sent for diagnostic confirmation to the World Organisation for Animal Health Reference Laboratory and the Food and Agriculture Organization of the United Nations Reference Center for Animal Influenza at the Istituto Zooprofilattico Sperimentale delle Venezie. Consistent with the laboratory test results provided by the submitting national veterinary laboratories, the presence of HPAI A(H5N1) virus was confirmed for 5 countries: Nigeria, Burkina Faso, Niger, Côte d’Ivoire, and Ghana. All samples positive for influenza A(H5N1) virus were sequenced by using Illumina MiSeq (San Diego, CA, USA) technology; complete coding sequences were obtained for 39 viruses, and the partial genome was obtained for 4 others (Table). To obtain consensus sequences later submitted to public databases (accession numbers in Table), we processed reads as described in Monne et al. (*9*). 

**Table:** Epidemiologic information for sequenced samples from poultry and wild birds positive for influenza A(H5N1) virus, West Africa*

Name	Sequenced genome	Collection date	Country, location	DB	Accession nos.
A/chicken/Ghana/15VIR5480-3/2015	Complete	2015 Jul 28	Ghana, Greater Accra	GB	KU971453–60
A/partridge/Ghana/15VIR5480-5/2015	Complete	2015 Jul 27	Ghana; Greater Accra	GB	KU971461–68
A/chicken/Ghana/15VIR5480-7/2015	Complete	2015 Jul 28	Ghana, Greater Accra	GB	KU971469–76
A/chicken/Ghana/15VIR5480-10/2015	Complete	2015 Aug 7	Ghana	GB	KU971397–04
A/chicken/Ghana/15VIR5480-12/2015	Complete	2015 Jul 27	Ghana, Greater Accra	GB	KU971405–12
A/chicken/Ghana/15VIR5480-14/2015	Complete	2015 Aug 7	Ghana, Greater Accra	GB	KU971413–20
A/duck/Ghana/15VIR5480-16/2015	Complete	2015 Jul 27	Ghana, Greater Accra	GS	EPI687323; EPI719449–55
A/chicken/Ghana/15VIR5480-18/2015	Complete	2015 Aug 7	Ghana, Greater Accra	GB	KU971421–28
A/chicken/Ghana/15VIR5480-22/2015	Complete	2015 Aug 7	Ghana, Greater Accra	GS	EPI687324; EPI719911–17
A/duck/Ghana/15VIR5480–24/2015	Complete	2015 Jull 27	Ghana, Greater Accra	GB	KU971429–36
A/chicken/Ghana/15VIR5480-26/2015	Complete	2015 Aug 7	Ghana, Greater Accra	GB	KU971437–44
A/chicken/Ghana/15VIR5480-27/2015	Complete	2015 Aug 7	Ghana, Greater Accra	GB	KU971445–52
A/chicken/Niger/15VIR2060-1/2015	Complete	2015 Apr 2	Niger, Maradi	GB	KU971301–08
A/chicken/Niger/15VIR2060-12/2015	Complete	2015 Apr 2	Niger, Maradi	GB	KU971309–16
A/chicken/Niger/15VIR2060-14/2015	Complete	2015 Apr 2	Niger, Maradi	GB	KU971317–24
A/chicken/Niger/15VIR2060-15/2015	HA	2015 Apr 2	Niger, Maradi	GB	KU971325
A/chicken/Niger/15VIR2060-5/2015	Complete	2015 Apr 2	Niger, Maradi	GB	KU971326–33
A/chicken/Niger/15VIR2060-6/2015	Complete	2015 Apr 2	Niger, Maradi	GB	KU971334–40
A/chicken/Niger/15VIR2060-7/2015	Complete	2015 Apr 2	Niger, Maradi	GB	KU971341–48
A/chicken/Niger/15VIR2060-8/2015	Complete	2015 Apr 2	Niger, Maradi	GB	KU971349–56
A/chicken/Ivory_Coast/15VIR2742-5/2015	Partial	2015 Apr 30	Côte d’Ivoire, Bouaké-Quartier Broukro	GB	KU971578–84
A/turtledove/Ivory_Coast/15VIR2742-7/2015	Complete	2015 Apr 30	Côte d’Ivoire, Bouaké-Quartier Broukro	GB	KU971585–92
A/duck/Ivory_Coast/15VIR2742-2/2015	Complete	2015 Apr 13	Côte d’Ivoire, Bouaké-Quartier Koko	GB	KU971562–69
A/chicken/Ivory_Coast/15VIR2742-3/2015	Complete	2015 Apr 13	Côte d’Ivoire, Bouaké-Quartier Koko	GB	KU971570–77
A/chicken/Burkina_Faso/15VIR1774-1/2015	Partial	2015 Mar 12	Burkina Faso, CPAVI in Ouagadougou	GB	KU971477–83
A/chicken/Burkina_Faso/15VIR1774-2/2015	Complete	2015 Mar 12	Burkina Faso, CPAVI in Ouagadougou	GB	KU971484–91
A/domestic_bird/Burkina_Faso/15VIR1774-22/2015	Complete	2015 Mar 10	Burkina Faso, Sanguiè Province	GB	KU971492–99
A/domestic_bird/Burkina_Faso/15VIR1774-23/2015	Complete	2015 Mar 10	Burkina Faso, Sanguiè Province	GB	KU971500–07
A/domestic_bird/Burkina_Faso/15VIR1774-24/2015	Complete	2015 Mar 10	Burkina Faso, Sanguiè Province	GB	KU971508–15
A/domestic_bird/Burkina_Faso/15VIR1774-25/2015	Complete	2015 Mar 10	Burkina Faso, Sanguiè Province	GB	KU971516–23
A/chicken/Burkina_Faso/15VIR1774-33/2015	Complete	2015 Mar 23	Burkina Faso, Koubri	GB	KU971524–31
A/chicken/Burkina Faso/15VIR1774-35/2015	Complete	2015 Mar 23	Burkina Faso, Koubri	GS	EPI584232; EPI719904–10
A/chicken/Burkina_Faso/15VIR1774-36/2015	Complete	2015 Mar 23	Burkina Faso, Koubri	GB	KU971532–39
A/chicken/Burkina_Faso/15VIR1774-37/2015	Complete	2015 Mar 12	Burkina Faso, CPAVI in Ouagadougou	GB	KU971540–47
A/chicken/Burkina_Faso/15VIR1774-38/2015	Partial	2015 Mar 12	Burkina Faso, CPAVI in Ouagadougou	GB	KU971548–53
A/chicken/Burkina_Faso/15VIR1774-4/2015	Complete	2015 Mar 12	Burkina Faso, CPAVI in Ouagadougou	GB	KU971554–61
A/chicken/Nigeria/15VIR339-1/2015	Complete	2015 Jan 2	Nigeria, Lagos State	GB	KU971593–00
A/chicken/Ghana/15VIR2588-10/2015	Complete	2015 May 8	Ghana, Greater Accra	GB	KU971357–64
A/chicken/Ghana/15VIR2588-11/2015	Complete	2015 May 4	Ghana, Greater Accra	GB	KU971365–72
A/chicken/Ghana/15VIR2588-4/2015	Complete	2015 May	Ghana, Greater Accra	GB	KU971373–80
A/chicken/Ghana/15VIR2588-5/2015	Complete	2015 May	Ghana, Greater Accra	GB	KU971381–88
A/chicken/Ghana/15VIR2588-8/2015	Complete	2015 May 8	Ghana, Greater Accra	GB	KU971389–96
A/chicken/Ghana/15VIR2588-9/2015	Complete	2015 May 4	Ghana, Greater Accra	GS	EPI632942; EPI719456–62

*CPAVI, Centre de Promotion de l’Aviculture Villageoise; DB, database; GB, GenBank; GS, The Global Initiative on Sharing All Influenza Data; HA, hemagglutinin.

We performed phylogenetic analyses for each genome segment by using PhyML 3.0 (*10*), incorporating a general time reversible model of nucleotide substitution with a gamma distribution of among-site rate variation (with 4 rate categories) and a subtree pruning and regrafting branch-swapping search procedure. The topology of the 8 phylogenetic trees shows that viruses collected from West Africa in 2015 belong to clade 2.3.2.1c and cluster separately from HPAI A(H5N1) viruses collected from West Africa during the 2006–2008 epidemic (Figure). Specifically, the analyzed viruses grouped with those that have been circulating in Eurasia since 2013 and showed the highest similarity with H5N1 subtype viruses collected in Europe and the Middle East from late 2014 through early 2015. As previously described for influenza A(H5N1) virus (*4*), the viruses from West Africa that we analyzed displayed the same genetic constellation of the A/Alberta/01/2014 virus; the polymerase basic protein 2 segment originated from a reassortment event with subtype H9N2. The hemagglutinin (HA) phylogenetic tree (Figure) shows that the viruses from West Africa constitute 2 main groups, here named WA1 and WA2, supported by high bootstrap values (>73%) and a genetic similarity of 98%–99.1%. WA1 is the most heterogeneous group (identity 98.7%–100%) and contains sequences from all affected countries in West Africa (Nigeria, Niger, Côte d’Ivoire, Burkina Faso, and Ghana). WA2 comprises sequences collected in April 2015 from Niger and Côte d’Ivoire only (identity 99.4%–100%) and clusters together with subtype H5N1 collected during January–March 2015 from wild birds in Europe (Bulgaria and Romania). Of note, viruses in the WA2 group are more closely related to those from Europe (similarity 99.30%–99.65%) than to those in the WA1 group (similarity 97.95%–99.12%), suggesting the occurrence of at least 2 independent introductions of subtype H5N1 in West Africa. Viruses in the WA1 and WA2 groups were isolated in 1 city in Niger and 1 city in Côte d’Ivoire, which suggests their possible co-circulation in the same geographic area.

**Figure:**
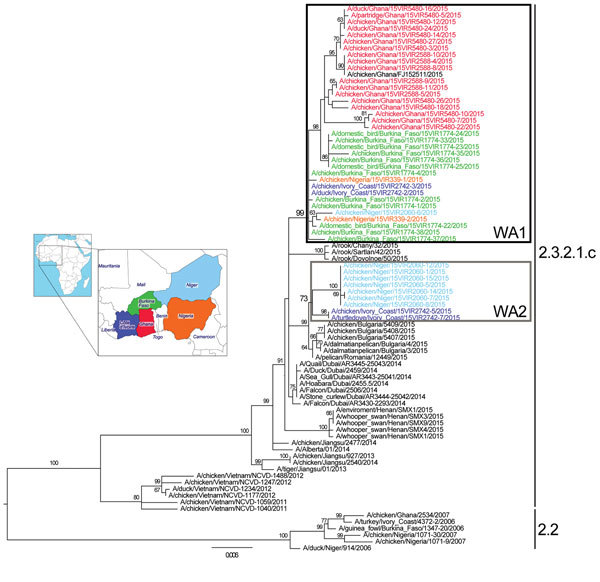
Maximum-likelihood phylogenetic tree of the hemagglutinin gene segment of highly pathogenic avian influenza (H5N1) viruses from West Africa. Strain colors indicate country of collection (inset). The 2 identified groups (WA1 and WA2) are indicated by boxes (black and gray, respectively). Clades are indicated at right; sequences from the 2006–2008 epidemic (clade 2.2) in West Africa were used as an outgroup. Numbers at the nodes represent bootstrap values >60%, obtained through a nonparametric bootstrap analysis that used 100 replicates. Scale bar indicates nucleotide substitutions per site.

As with the HA gene, we identified the 2 West Africa groups in all the other phylogenies (supporting bootstrap values >74%), except for the tree of the nonstructural gene segment, in which WA2 does not form a monophyletic group. Unfortunately, only the HA gene segment of the viruses from Europe that clusters with the WA2 group is available in the public database, making the source of the internal genes of the WA2 viruses impossible to trace.

The analysis of molecular markers indicates that all viruses showed mutations D94N (except for A/chicken/Ghana/5480-14/2015), S133A, and S155N (H5 numbering) in the HA protein; these mutations have been shown to increase virus binding to α2,6 sialic acid (*11*). In addition, the analysis of internal proteins identified a mutation associated with enhanced replication efficiency (NP N319K) (*11*) in all WA2 viruses from Niger. Moreover, the alternative reading frame of the polymerase basic protein 1 of the WA2 viruses is truncated (57 aa long), as it is in the Asian and European progenitors. This truncation is common among influenza A viruses of mammals and in HPAI A(H5N1) viruses, and it has been associated with increased virulence in mammals (*12*).

## Conclusions

We demonstrated that a reassortant HPAI A(H5N1) clade 2.3.2.1c virus was responsible for infections in 5 West Africa countries. The influenza (H5N1) viruses from West Africa show a close phylogenetic relationship with the HPAI A(H5N1) viruses identified in Europe and the Middle East during late 2014–2015, indicating a Eurasian origin of their progenitors. The route of introduction of this virus is difficult to establish because West Africa offers wintering sites for wild birds coming from the southern Russian regions, Europe, and western Asia (*13*), and it imports live birds from countries in Europe and Asia (*14*).

As with previous epidemics (2006–2008), when distinct introductions and multiple reassortment events were identified (*3*,*15*), we were able to detect the co-circulation of 2 distinct genetic clusters in Côte d’Ivoire and Niger, which suggests that there might have been at least 2 separate introductions into West Africa. However, the limited amount of genetic data available makes it impossible to pinpoint how these viruses entered the continent and spread so widely, and it is not easy to determine the exact number of introductions and where they have occurred in West Africa. Additional virus data from affected countries would help elucidate the epidemiology and the evolution of this virus in this part of the continent.

Of note, all the viruses from West Africa display the same genetic constellation of a strain (A/Alberta/01/2014) isolated from a human, a Canada resident who had returned from China. These viruses contain mutations that have been described as being associated with an enhanced binding affinity for α2,6 sialic acid or with increased virulence in mammals.

As during the 2006–2008 HPAI A(H5N1) epidemics, West Africa countries are again facing devastating economic and social consequences from these infections. It is imperative for regional and international organizations to join forces in generating and making available detailed genetic and epidemiologic information that can be used to better trace the spread and evolution in West Africa of influenza A(H5N1) virus and to provide input for informed decisions on control measures and resource allocation.

Technical Appendix Global Initiative on Sharing All Influenza Data accession numbers, submitting laboratories, and authors of the sequences used in study of genetically different highly pathogenic avian influenza A(H5N1) viruses in West Africa, 2015.
