# Supplementary material for: Genetically Different Highly Pathogenic Avian Influenza A(H5N1) Viruses in West Africa, 2015
Source: Emerg Infect Dis. 2016 Dec;22(12):2132–6. doi: 10.3201/eid2212.160578 (PMC5189143; doi:10.3201/eid2212.160578)
Supplement: Technical Appendix — Global Initiative on Sharing All Influenza Data accession numbers, submitting laboratories, and authors of the sequences used in study of genetically different highly pathogenic avian influenza A(H5N1) viruses in West Africa, 2015. [file 16-0578-Techapp-s1.pdf]

Article DOI: http://dx.doi.org/10.3201/eid2212.160578

Genetically Different Highly Pathogenic Avian Influenza A(H5N1) Viruses in West Africa, 2015

Technical Appendix

Technical Appendix Table. Global Initiative on Sharing All Influenza Data (GISAID) accession numbers, submitting laboratories, and authors of the sequences used in study of genetically different highly pathogenic avian influenza A(H5N1) viruses in West Africa, 2015*

Segment ID	Segment	Country	Collection date	Isolate name	Originating Lab	Submitting Lab	Authors
EPI592605	HA	Bulgaria	2015 Jan 23	A/dalmatian pelican/Bulgaria/ 4/ 2015	NDRVMI (National Diagnostic and Research Veterinary Medical Institute)	Animal and Plant Health Agency (APHA)	Thomas, S; Seekings, A; Essen, S; Manvell, R; Goujgoulova, G; Oreshkova, L; Banks, J; Brown, I
EPI592416	HA	Bulgaria	2015 Jan 23	A/dalmatian pelican/Bulgaria/ 3/ 2015	NDRVMI (National Diagnostic and Research Veterinary Medical Institute)	Animal and Plant Health Agency (APHA)	Thomas, S; Seekings, A; Essen, S; Manvell, R; Goujgoulova, G; Oreshkova, L; Banks, J; Brown, I
EPI594492	HA	Bulgaria	2015 Jan 30	A/chicken/ Bulgaria/5409/15	NDRVMI (National Diagnostic and Research Veterinary Medical Institute)	Animal and Plant Health Agency (APHA)	Thomas, S; Seekings, A; Essen, S; Manvell, R; Goujgoulova, G; Oreshkova, L; Banks, J; Brown, I
EPI594491	HA	Bulgaria	2015 Jan 30	A/chicken/ Bulgaria/5408/15	NDRVMI (National Diagnostic and Research Veterinary Medical Institute)	Animal and Plant Health Agency (APHA)	Thomas, S; Seekings, A; Essen, S; Manvell, R; Goujgoulova, G; Oreshkova, L; Banks, J; Brown, I
EPI594490	HA	Bulgaria	2015 Jan 30	A/chicken/ Bulgaria/5407/15	NDRVMI (National Diagnostic and Research Veterinary Medical Institute)	Animal and Plant Health Agency (APHA)	Thomas, S; Seekings, A; Essen, S; Manvell, R; Goujgoulova, G; Oreshkova, L; Banks, J; Brown, I
EPI559770	HA	China	2015 Jan 04	A/whooper swan/Henan/ SMX3/ 2015(H5N1)	CAS Key Laboratory of Pathogenic Microbiology and Immunology, Institute of Microbiology, Chinese Academy of Sciences	Institute of Microbiology, Chinese Academy of Sciences	Not applicable
EPI559727	HA	China	2015 Jan 04	A/whooper swan/Henan/ SMX1/ 2015(H5N1)	CAS Key Laboratory of Pathogenic Microbiology and Immunology, Institute of Microbiology, Chinese Academy of Sciences	Institute of Microbiology, Chinese Academy of Sciences	Not applicable
EPI559910	HA	China	2015 Jan 05	A/whooper swan/Henan/ SMX9/ 2015(H5N1)	CAS Key Laboratory of Pathogenic Microbiology and Immunology,	Institute of Microbiology, Chinese Academy of Sciences	Not applicable

Segment ID	Segment	Country	Collection date	Isolate name	Originating Lab	Submitting Lab	Authors
EPI559789	HA	China	2015 Jan 05	A/whooper swan/Henan/SMX4/2015(H5N1)	Institute of Microbiology, Chinese Academy of Sciences CAS Key Laboratory of Pathogenic Microbiology and Immunology, Institute of Microbiology, Chinese Academy of Sciences	Institute of Microbiology, Chinese Academy of Sciences	Not applicable
EPI560202	HA	China	2015 Jan 05	A/enviroment/Henan/SMX1/2015(H5N1)	Institute of Pathogen Biology, Taishan Medical College	Institute of Microbiology, Chinese Academy of Sciences	Shi, Weifeng; Bi, Yuhai
EPI594560	HA	Romania	2015 Mar 26	A/pelican/Romania/12449/2015	Institute for Diagnosis & Animal Health (IDAH)	Animal and Plant Health Agency (APHA)	Thomas, S; Puranik, A; Londt, B; Essen, S; Manvell, R; Onita, I; Neicut, A; Cioranu, R; Motiu, R; Banks, J; Brown, I
EPI631928	HA	Russian Federation	2015 May 07	A/rook/Sartlan/42/2015	State Research Center of Virology and Biotechnology Vector	State Research Center of Virology and Biotechnology Vector	Ivan, Susloparov; Vasily, Marchenko; Natalya, Goncharova; Andrey, Shipovalov; Alexander, Durymanov; Tatyana, Ilyicheva; Alexander, Ryzhikov
EPI631920	HA	Russian Federation	2015 May 07	A/rook/Dovolnoe/50/2015	State Research Center of Virology and Biotechnology Vector	State Research Center of Virology and Biotechnology Vector	Ivan, Susloparov; Vasily, Marchenko; Natalya, Goncharova; Andrey, Shipovalov; Alexander, Durymanov; Tatyana, Ilyicheva; Alexander, Ryzhikov
EPI631912	HA	Russian Federation	2015 May 07	A/rook/Chany/32/2015	State Research Center of Virology and Biotechnology Vector	State Research Center of Virology and Biotechnology Vector	Ivan, Susloparov; Vasily, Marchenko; Natalya, Goncharova; Andrey, Shipovalov; Alexander, Durymanov; Tatyana, Ilyicheva; Alexander, Ryzhikov
EPI623563	HA	United Arab Emirates	2014 Dec 01	A/Duck/Dubai/2459/2014		Friedrich-Loeffler-Institut	Chen, H.; Chan, KH. ; Wong, PC. ; Woo, C.Y.P.
EPI623571	HA	United Arab Emirates	2014 Dec 07	A/Falcon/Dubai/2506/2014		Friedrich-Loeffler-Institut	Chen, H.; Chan, KH. ; Wong, PC. ; Woo, C.Y.P.
EPI603561	HA	United Arab Emirates	2014 Dec 07	A/Sea Gull/Dubai/AR3443–25041/2014		Friedrich-Loeffler-Institut	Naguib, M.M.; Wernery, U.; Harder, T.
EPI603577	HA	United Arab Emirates	2014 Dec 07	A/Quail/Dubai/AR3445–25043/2014		Friedrich-Loeffler-Institut	Naguib, M.M.; Wernery, U.; Harder, T.
EPI603569	HA	United Arab Emirates	2014 Dec 07	A/Stone curlew/Dubai/AR3444–25042/2014		Friedrich-Loeffler-Institut	Naguib, M.M.; Wernery, U.; Harder, T.
EPI603553	HA	United Arab Emirates	2014 Nov 17	A/Falcon/Dubai/AR3430–2293/2014		Friedrich-Loeffler-Institut	Naguib, M.M.; Wernery, U.; Harder, T.

Segment ID	Segment	Country	Collection date	Isolate name	Originating Lab	Submitting Lab	Authors
EPI623555	HA	United Arab Emirates	2014 Nov 30	A/Hoabara/ Dubai/ 2455.5/2014		Friedrich-Loeffler-Institut	Chen, H.; Chan, KH. ; Wong, PC. ; Woo, C.Y.P.
EPI643194	HA	Ghana	2015 Jan 01	A/chicken/ Ghana/ FJ152511/2015	University of Ghana	Crick Worldwide Influenza Centre	Not applicable
EPI556504	HA	Nigeria	2015 Jan 01	A/chicken/ Nigeria/ 15VIR339–2/2015	National Veterinary Research Institute	Istituto Zooprofilattico Sperimentale Delle Venezie	Joannis, T.; Ahmed, M.; Meseko, C.; Shittu, I.; Solomon, P.; Luka, P.; Olorunshola, B.; Tassoni, L.; Schivo, A.; Ormelli, S.; Monne, I.
EPI425248	HA	Vietnam	2012 Jan 30	A/chicken/ Vietnam/NCVD-1177/2012	National Centre of Veterinary Diagnostics	Centers for Disease Control and Prevention	Not applicable
EPI425376	HA	Vietnam	2012 Feb 17	A/duck/Vietnam/ NCVD-1234/2012	National Centre of Veterinary Diagnostics	Centers for Disease Control and Prevention	Not applicable
EPI425472	HA	Vietnam	2012 Feb 16	A/chicken/ Vietnam/NCVD-1247/2012	National Centre of Veterinary Diagnostics	Centers for Disease Control and Prevention	Not applicable
EPI425616	HA	Vietnam	2012 Mar 02	A/chicken/ Vietnam/NCVD-1488/2012	National Centre of Veterinary Diagnostics	Centers for Disease Control and Prevention	Not applicable
EPI425787	HA	Vietnam	2011 Sep 07	A/chicken/ Vietnam/NCVD-1040/2011	National Centre of Veterinary Diagnostics	Centers for Disease Control and Prevention	Not applicable
EPI425827	HA	Vietnam	2011 Sep 08	A/chicken/ Vietnam/NCVD-1059/2011	National Centre of Veterinary Diagnostics	Centers for Disease Control and Prevention	Not applicable
EPI500771	HA	Canada	2014 Jan 03	A/Alberta/01/ 2014	Provincial Laboratory of Public Health for Southern Alberta	Public Health Agency of Canada (PHAC)	Li, Yan; Bastien, Nathalie; Fonseca, Kevin; Tipples, Graham; Pabbaraju, Kanti; Tellier, Raymond; Wong, Sallene; Tang, Julian W.; Drews, Steven J.

*We acknowledge the authors and the originating and submitting laboratories of the sequences from GISAID's EpiFlu Database on which this research is based. All submitters of data can be contacted directly through www.gisaid.org.
